# Enhanced Stability of O/W Pickering Emulsions Driven by Interfacial Adsorption of Whey Protein Nanogels

**DOI:** 10.3390/foods15010009

**Published:** 2025-12-19

**Authors:** Zhaoshuo Yu, Fangzhou He, Lijing Ke, Jean-Christophe Jacquier

**Affiliations:** 1 Institute of Food and Health, School of Agriculture and Food Science, University College Dublin, Belfield, D04 C1P1 Dublin, Ireland; zhaoshuo.yu1@ucdconnect.ie; 2 School of Food Science and Nutrition, University of Leeds, Leeds LS2 9JT, UK; dslm1835@leeds.ac.uk (F.H.); l.ke@leeds.ac.uk (L.K.)

**Keywords:** flow behaviour, physical stability, Pickering emulsion, surface coverage, theoretical calculations, whey protein nanogel

## Abstract

Whey protein is valued for its health and emulsifying benefits, yet its intrinsic instability limits its effectiveness as an emulsifier under food processing conditions. To address the need for physically stable emulsions, this study developed O/W Pickering emulsions stabilised by nanogel WPI (GWEs) and investigated their stability under common food processing conditions, including thermal treatment, pH adjustment, and cold storage. For comparison, emulsions stabilised by non-heated (NWEs) and heat-treated WPI (HWEs) were also prepared. The results showed that while the oil droplet size of GWEs (12.2 ± 1.16 µm) was comparable to NWEs (13.6 ± 0.26 µm), HWEs exhibited significantly larger droplets (18.0 ± 0.16 µm). GWEs demonstrated the highest protein adsorption at the oil–water interface (68.7%). TEM further revealed that whey nanogels achieved nearly full monolayer coverage of oil droplets. By contrast, only partial protein coverage and exposed interfaces were observed in NWEs and HWEs. Additionally, GWEs exhibited superior stability under food processing conditions, with minimal changes in emulsion capacity, droplet size, viscosity, and flow behaviour when subjected to heat (up to 90 °C), acidification (pH down to 3), and storage for up to 3 days, confirming the potential of nanogel WPI as an advanced stabiliser in emulsion-based formulations.

## 1. Introduction

In recent years, whey protein has become a highly valuable ingredient due to its unique functional properties and high nutritional value [1]. Within emulsion systems, whey protein serves as an effective emulsifier, quickly diffusing and adsorbing at the oil–water (O/W) interface [2]. However, whey proteins often become unstable under typical food processing conditions, with factors like high temperatures, acidic pH, and extended storage leading to protein denaturation and aggregation, which can ultimately reduce emulsion stability [3].

An alternative is solid protein particle-based stabilisation, particularly Pickering emulsions. Pickering emulsions stabilised by solid particles offer superior physical stability compared with traditional protein emulsifier, which may be related to the barrier effects created by protein particles in Pickering emulsions [4,5]. Among them, whey protein micro-/nanogels have been fabricated as an attractive option for Pickering emulsion stabilisation [6]. This shows that whey protein microgels can form relatively stable emulsions across a wide pH range and ionic strengths by forming continuous particle networks at the O/W interface, thereby helping prevent droplet coalescence [7]. Modulating the particle size of these gels, such as via Ca^2+^ crosslinking, has not only enabled the preparation of high-internal-phase Pickering emulsions with prolonged storage stability [8], but has also expanded their role as efficient nutrient delivery platforms, enhancing the colloidal stability, controlled release, and bio-accessibility of lipophilic bioactives such as β-carotene and CoQ10 [9]. Despite advances in formulating whey protein gels for Pickering stabilisation, their adsorption behaviour and arrangement at the oil–water interface remain insufficiently understood, leaving a key mechanistic gap to be addressed.

Current mechanistic research has primarily revolved around quantifying protein adsorption at the O/W interface as an indicator of emulsifying ability, while key aspects such as protein surface coverage and arrangement have received relatively limited attention. In whey protein-stabilised emulsions, protein unfolding at the interface can increase surface load and coverage, but it does not inherently ensure a uniform monolayer or a full droplet coverage, as coalescence beyond the limited coalescence regime halts further coverage [7]. On the other hand, Pickering particles are not always evenly distributed across the interface. For example, when protein particle content exceeds 1%, a protein multilayer or aggregates at the interface may be formed [10]. When whey protein particles form multilayer or aggregates on droplet surfaces, such structures can reduce the maximum achievable surface load and compromise the physical barrier properties essential for emulsion stability [11,12]. In contrast, close and uniform protein packing has been associated with higher colloidal stability and extended shelf life of emulsions [13]. These limitations elucidate the need for protein-based stabilisers capable of achieving dense and full surface coverage of emulsion droplets.

Our previous research [14] developed negatively charged, submicron-sized nanogel WPI via a limited calcium–heat treatment, without the need for homogenisation or mechanical shearing. Compared with nanogels prepared by heat-induced gelation followed by homogenisation, or by heat-induced gelation combined with subsequent calcium crosslinking and homogenisation [9], this rapid (30 min) approach directly yields nanogels rather than macrogels, enabling controlled particle size without additional size-reduction steps [14]. Furthermore, this nanogel WPI demonstrated excellent stability under typical food processing conditions, such as high ionic strength, shear force, heat treatment, and a wide pH range [14], serving as a potential candidate for stabilising emulsions.

Thus, this study aims to develop O/W Pickering emulsions stabilised by whey protein nanogels. To compare the physical stability, three types of whey protein were used to prepare emulsions: non-heated WPI and heat-treated WPI, which act as more traditional emulsifiers, and nanogel WPI, which serve as particle-based emulsifiers. The stability of each emulsion was then evaluated by measuring key quality parameters, such as oil droplet size, emulsion capacity, and emulsion viscosity, under typical food processing conditions. Interfacial protein properties including protein adsorption at the oil–water interface, surface loading, and surface coverage were also examined together with theoretical modelling and calculations to provide insights into the mechanisms influencing emulsion stability.

## 2. Materials and Methods

### 2.1. Materials

Whey protein isolate (WPI) (93% protein; 0.79% calcium) was obtained from Fonterra Cooperative Group Limited (Auckland, New Zealand). Rapeseed oil was purchased from the local supermarket. Calcium chloride dihydrate, sodium hydroxide, and hydrochloric acid were all purchased from Sigma Aldrich (Wicklow, Ireland). All reagents used were of analytical grade.

### 2.2. Fabrication of Non-Heated, Heat-Treated and Nanogel WPI

Nanogel WPI was prepared according to our previously published study [14]. Briefly, an 8% (*w*/*w*) protein solution was prepared by dissolving 8.6 g of WPI in 91.4 g of deionized water. The solution was then stirred on a magnetic stirrer (IKA, Staufen, Germany) for two hours at room temperature and subsequently stored at 4 °C overnight to ensure the complete hydration of the protein. After hydration, the solution was centrifuged at 8000 rpm for 30 min at 20 °C to remove insoluble substances, and the pH of the supernatant was adjusted to 6.9 to obtain non-heated WPI. Heat-treated WPI was prepared by heating the protein supernatant at 90 °C for 30 min, followed by rapid cooling to room temperature. For the preparation of nanogel WPI, calcium chloride was added to the non-heated WPI solution to achieve a final concentration of 6.0 mM. The resulting solution was subsequently heated at 90 °C for 30 min and rapidly cooled to room temperature to induce the formation of nanogels for further use [14].

### 2.3. Preparation of Emulsions Stabilised by Non-Heated WPI, Heat-Treated WPI, and Nanogel WPI, Respectively

Based on established emulsion preparation protocols [15,16,17], emulsions stabilised by non-heated WPI (NWEs), heat-treated WPI (HWEs), and nanogel WPI (GWEs) were prepared by mixing rapeseed oil (10%, *v*/*v*) with the diluted protein aqueous solution (final protein concentration of 1.0%, *w*/*v*) using a high-speed homogenizer (T-25 digital Ultra Turrax, IKA-Werke, Staufen im Breisgau, Germany) operating at 12,000 rpm for 2 min. Pauses were taken every 30 s to prevent overheating.

### 2.4. Measurements of Particle Size and Specific Surface Area (A)

The oil droplet size and specific surface area (*A*) of emulsions was determined using a laser diffraction particle size analyser (Mastersizer 3000, Malvern Instruments, Malvern, UK). Refractive indices input of dispersed rapeseed oil and deionized water were taken as 1.47 and 1.33, respectively. The oil droplet diameter was measured as the volume-weighted mean diameter (D[4, 3]) and surface area moment mean (D[3, 2]).

Specific oil droplet surface area (*A* in m^2^/kg) was calculated using Equation (1) according to Zhang et al. [18]:

(1) $$A=\frac{6}{\rho \times D[3,2]}$$

where ρ is the density of rapeseed oil used in the emulsion (0.9 kg/L).

### 2.5. Dynamic Light Scattering (DLS) Determination

ζ-potential analysis of the emulsions was conducted using DLS with a Zetasizer Nano ZS (Malvern Instruments Co., Ltd.). at 25 °C. All emulsions were diluted 100-fold prior to measurement with deionized water to avoid multiple light scattering [19].

### 2.6. Morphological Observation

To conduct morphological observations of emulsion droplets, five microlitres of the emulsion samples were deposited onto a carbon-coated copper grid. The samples were then negatively stained using 2% uranyl acetate, utilising the side blot method for enhanced contrast and detail in the microscopic images. These prepared samples were examined using a transmission electron microscope (FEI, Wellington, FL, USA), which operated at a voltage of 80 keV and utilised a tungsten filament [20,21].

### 2.7. Protein Adsorption and Surface Load

The protein content at the oil–water interface was analysed using a UV–vis spectrophotometer (UV Mini 1240, Shimadzu, Kyoto, Japan) to determine the adsorbed protein and surface load based on a previous approach [10] with some minor modifications. Briefly, the freshly made emulsion was centrifuged for 30 min under 3000× *g* at 4 °C within a temperature-regulated centrifuge (Avanti J-15R, Beckman Coulter, Brea, CA, USA) to separate the un-adsorbed protein from the emulsion. The aqueous protein phase was then collected carefully using a syringe after centrifugation. Before testing, the collected protein phase was diluted to achieve a protein concentration of approximately 1.0 g/L. For non-heated WPI and heat-treated WPI, which exhibit minimal light scattering interference, protein concentration was directly determined by measuring absorbance at 280 nm and an extinction coefficient ε_(280)_ = 1.046 L/cm/g [22]. By contrast, nanogel WPI displays colloidal properties and stronger scattering effects. Therefore, its protein concentration was determined using a calibration curve relating 280 nm absorbance to nanogel WPI protein concentration (y = 2.0888x + 0.0566; R^2^ = 0.999).

Then, according to Qin et al. [17], protein adsorption and surface load Γs (mg/m^2^) were calculated by Equations (2) and (3), respectively:

(2) $$Adsorption\%=\frac{\left.C_{i}-C_{f}\right.}{C_{i}}\times 100\%$$

(3) $$\Gamma s=\frac{\left.C_{i}-C_{f}\right.}{A}\times 1000$$

where *C_i_* represents the total protein concentration of the emulsion (10 g/L). *C_f_* refers to the un-adsorbed protein concentration in the aqueous phase of the emulsions after centrifugation, and *A* is the specific surface area (m^2^/kg) of the emulsion droplets determined by Equation (1).

### 2.8. Theoretically Estimation of Surface Coverage (Cs)

According to previous studies by Binks et al. [23] and Araiza-Calahorra et al. [10], the surface coverage of proteins on the oil droplet can be theoretically estimated (assuming all of the protein is at the oil droplet interface) using Equation (4):

(4) $$Cs=\frac{{D[3,2]\cdot m}_{p}}{4\cdot V_{o}\;{\cdot \;\rho }_{p}\cdot d_{p}}$$

where D[3, 2] represents the mean oil droplet diameter, m*_p_* represents the mass of the protein phase, which is 1.0 g for a 100 mL emulsion. *Vo* denotes the volume of the oil phase, given as 10 mL for a 100 mL emulsion. $\rho_{p}$ is the specific gravity of the protein, specified as 1.37 g/cm^3^ [24] and *d_p_* refers to the protein or protein particle diameter. The *d_p_* of heat-treated WPI and nanogel WPI was determined using DLS, and found to be 83.0 nm and 275.0 nm, respectively. In this model, the size of non-heated WPI was represented by the diameter of β-lactoglobulin, the main component of whey protein, taken to be 3.5 nm [25].

Under the assumption that the spherical protein particles form a honeycomb-like pattern on the surface of the oil droplets (hexagonal packing), the maximum theoretical surface coverage is 0.9.

### 2.9. Emulsion Capacity (EC)

The EC was measured according to the method described by Cano-Medina et al. [26] after centrifugation at 1400× *g* for 15 min according to Equation (5):

(5) $$EC\%=\frac{Volume\;of\;the\;emulsified\;layer}{Total\;volume\;of\;emulsion}\times 100\%$$

### 2.10. Viscosity Determination

The viscosity properties of emulsions were measured at 25 °C with a Physica MCR-301 rheometer (Anton Paar, Graz, Austria) using steel parallel plates (40 mm diameter; 0.100 mm gap). Before each measurement, the emulsion was deposited on a plate for 5 min to allow the temperature to equilibrate. The shear rate of the steady-state flow sweep was measured from 1 to 100 s^−1^.

Flow behaviour was measured according to the power-law model (Ostwald–de Waele model) Equation (6).

η = K · γ^n−1^ (6)

where *η* is the viscosity (mPa·s), *K* is the consistency index (mPa·s^n^), γ is the shear rate (s^−1^) and n the flow behaviour index (dimensionless).

### 2.11. Physical Stability of Emulsions

To determine the physical stability of emulsions after typical food processing treatments, heat stability, pH stability, and storage stability experiments were carried out.

*Heat stability*. The fresh emulsions were incubated in a water bath at 25 °C, 60 °C, and 90 °C for 30 min. After each treatment, the mean droplet size (D[4, 3]), emulsion capacity, and viscosity of the emulsions were measured.

*pH stability*. The pH of fresh emulsions was adjusted to 7.0, 5.0, and 3.0, with 1 M hydrochloric acid or sodium hydroxide. After pH adjustment, the mean droplet size (D[4, 3]), emulsion capacity, and viscosity of the emulsions were measured.

*Storage stability*. The fresh emulsions were placed at 4 °C for up to 3 days. After cold storage, the mean droplet size (D[4, 3]), emulsion capacity, and viscosity of the emulsions were measured.

### 2.12. Data Analysis

The experiments were carried out in triplicate. Statistical analysis was conducted using one-way analysis of variance (ANOVA) with Tukey’s multiple comparison tests, with significant differences between sample data indicated at *p* < 0.05. Results were then presented as means ± standard deviations.

## 3. Results and Discussion

### 3.1. Characteristics of NWE, HWE, and GWE

In all three emulsions, the oil phase and protein concentration were maintained at 10% (*v*/*v*) and 1.0% (*w*/*v*), respectively. The main characteristics of these emulsions were measured and compared in Table 1. Regarding droplet size, the D[3, 2] values indicated that NWE and GWE had similar sizes of 12.2 μm and 13.6 μm, respectively, which were significantly smaller than HWE’s droplet size of 18.0 μm. A similar trend was observed with the D[4, 3] values (26.5 μm for NWE, 27.8 μm for GWE, and 37.7 μm for HWE). These results indicate that HWE had the largest droplets among the three, which corresponded to the smallest specific surface area. Those results of NWE and HWE are similar with previous studies. For example, at the same protein concentration of 1%, the D[3, 2] values of non-heated WPI-stabilised emulsions reported by Kaade et al. [27] and Hebishy et al. [28] were 13.9 ± 0.55 μm and 16.7 ± 0.37 μm, respectively, which are comparable to that of NWE in this study.

In addition, it seems that at the same protein concentration of 1%, emulsions stabilised by heat-treated WPI tend to have larger droplets than those stabilised by non-heated WPI [29]. Sarkar et al. [30] and Destribats et al. [7] reported D[4, 3] values exceeding 40 μm for emulsions stabilised with heated WPI, slightly higher than the HWE results observed here. This may be attributed to the reduced capacity of heat-treated WPI to stabilise the newly created interfaces during the homogenization process, which finally generated larger emulsion droplets [31], and reduced specific surface area. Interestingly, although GWE was prepared using nanogel WPI that had also undergone heat treatment, it still exhibited relatively small droplet sizes, comparable to those of the non-heated WPI emulsion.

NWE exhibited the highest ζ-potential absolute value at −56.2 mV, followed by HWE (−52.1 mV) and GWE (−41.7 mV). This is consistent with a previous study by Du et al. [32] who reported ζ-potential values of approximately −50 mV for emulsions based on non-heated WPI. The ζ-potential of protein-based emulsions primarily reflects the surface charge of proteins adsorbed at the oil–water interface. In this study, NWE, HWE, and GWE were stabilised by non-heated WPI, heat-treated WPI, and nanogel WPI, respectively. Heat treatment can induce structural unfolding of proteins and the loss of negatively charged functional groups [32], resulting in a decrease in the absolute ζ-potential value in HWE. In the case of GWE, nanogel WPI was formed through a combination of heat treatment and calcium ions. Calcium ions can further reduce the electrostatic repulsion between negatively charged protein molecules [14] and can explain why GWE exhibited lower absolute ζ-potential than the others. Despite the differences, the absolute ζ-potential values for all three emulsions were well above 30 mV, a value established to confer relatively good colloidal stability [31].

### 3.2. Protein Adsorption and Surface Load

As shown in Table 1, GWE exhibited the highest adsorption efficiency, reaching nearly 70%, with much lower values obtained for HWE and NWE. This is consistent with previous studies reporting that within non-heated whey protein-based emulsion systems, around 80% of the protein typically remain un-adsorbed in the aqueous phase while heat treatment can enhance protein adsorption at the O/W interface [33]. The adsorption value of 30% observed for HWE in this study closely matches published values [30].

Similarly, GWE demonstrated the highest surface protein load, with 13.6 mg/m^2^ of nanogel on the droplet surface, compared to only 4.0 mg/m^2^ for non-heated WPI and 8.1 mg/m^2^ for heat-treated WPI at the interface. This aligns with published studies, which reported a surface protein load of approximately 3.0 mg/m^2^ for non-heated whey protein-stabilised emulsions [28] at the same protein concentration (1.0% *w*/*v*), while emulsions stabilised with heat-treated WPI exhibited surface loads around 7.5 mg/m^2^ [34].

The difference in interfacial adsorption and surface load seen here between emulsion samples may directly impact their emulsion capacity (Table 1) as GWE demonstrated a markedly enhanced capacity, with more than 3-fold increase compared to the other two samples. This may be because larger protein particles, such as nanogel WPI, have higher anchoring energy and greater resistance to Brownian motion, enabling them to form more stable membranes at the oil–water interface, thereby enhancing the physical stability of the emulsion [14]. By contrast, smaller protein particles, such as non-heated proteins, are more susceptible to interference from Brownian motion, resulting in insufficient anchoring at the interface [35], resulting in lower emulsion capacity.

### 3.3. Microstructures of Emulsions

**Figure 1:**
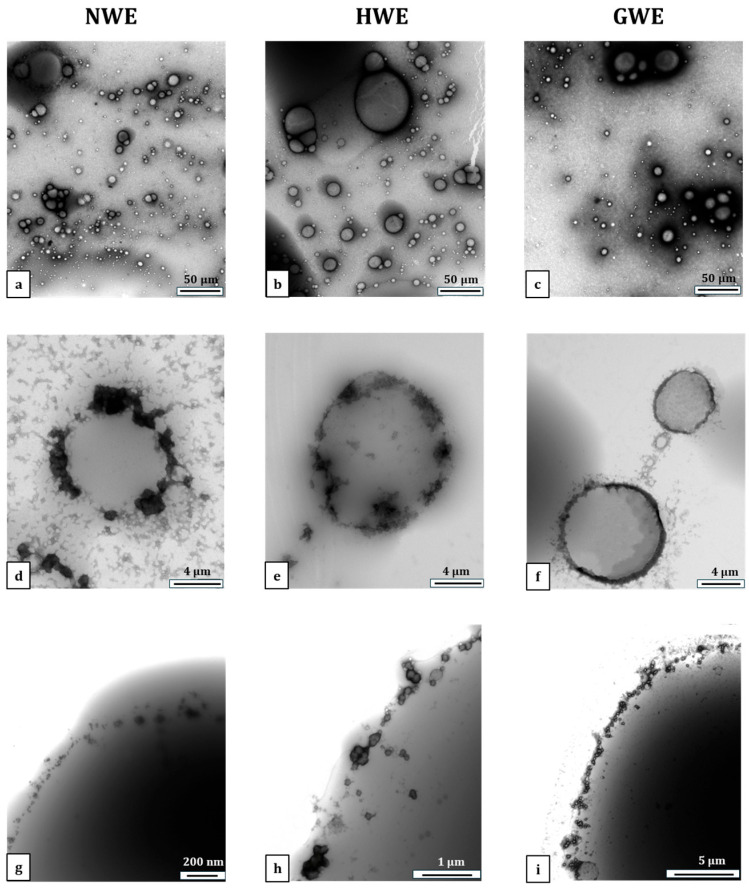
TEM images of emulsions. Panels (**a**–**c**) show overall views of the emulsions, (**d**–**f**) individual emulsion droplets, and (**g**–**i**) interfaces of emulsion droplets. The first column represents NWE, the second column represents HWE, and the third column is GWE. Magnifications are shown in bars.

TEM images, as shown in Figure 1, further confirm the differences in interface coverage, and protein adsorption and aggregation among the three emulsions. In Figure 1a–c, GWE exhibits a more uniform distribution of spherical droplets, with relatively smaller and more consistently sized particles compared to HWE that contains larger and less uniform droplets. In contrast, NWE displays greater polydispersity and noticeable droplet aggregation. Figure 1d–f revealed that the surface of GWE was covered by a relatively dense protein film, while oil surface exposure was observed for HWE and NWE. In the case of NWE (Figure 1d), non-heated WPI aggregated intensely, forming large-scale clusters. In spite of less aggregation of heat-treated WPI in HWE, there was still visible oil exposure at the interface (Figure 1e). This is in contrast to what can be observed in the case of GWE (Figure 1f) where near-complete coverage of the oil droplet by a thin protein layer can be seen.

High-magnification images (Figure 1g–i) further confirm these differences in interfacial organisation. In NWE, non-heated WPI appeared sparsely and irregularly adsorbed on the interface, forming a discontinuous and loose layer. This is in line with published TEM studies of emulsions stabilised by 1.0% non-heated WPI, where a relatively low protein adsorption was also observed at the O/W interface [28]. Heat-treated WPI showed a relatively enhanced protein adsorption compared to non-heated WPI, with more defined particles present at the interface. However, a continuous and dense protein film was still not achieved, in agreement with earlier studies employing both TEM and cryo-SEM, which revealed partial protein aggregates and exposure at the interface [10]. GWE, however, presented a relatively uniform and full nanogel layer at the droplet surface, suggesting possibly stronger interfacial anchoring and better barrier formation.

As shown in Table 1, the surface coverage of GWE was an ideal 0.9, signifying that 90% of the average oil droplet’s surface was covered. This high coverage aligns with the model’s assumption that adsorbed particles are monodispersed at the oil–water interface in a hexagonal close packing arrangement, the densest spatial configuration of spheres on a surface [10,23], which theoretically should yield a saturation surface coverage of 0.9 [12]. Conversely, the Cs values for NWE and HWE were significantly greater than 1.0, indicating that the interfacial coverage by non-heated or heat-treated WPI may involve excess un-adsorbed proteins in the system or multilayer adsorption, or have areas of exposure due to overlapping or insufficient coverage [10].

### 3.4. Rheological Characteristics

**Figure 2:**
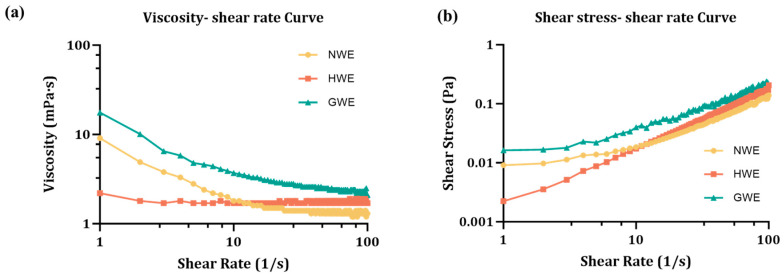
Rheological behaviour of emulsions. (**a**) Viscosity–shear rate curve. (**b**) Shear stress–shear rate curve.

As shown in Figure 2a, both NWE and GWE exhibited characteristic shear-thinning non-Newtonian behaviour, where viscosity decreased significantly with increasing shear stress. By contrast, HWE maintained an almost constant viscosity across the shear rate range, indicating Newtonian behaviour. This result aligns with previous reports by Zamani et al. [5] who showed that emulsions prepared with 1% non-heated WPI exhibited shear-thinning behaviour, while Çakır-Fuller [33] reported that the rheological behaviour of heat-treated WPI-based emulsions was generally concentration-dependent. Specifically, heat-treated WPI-stabilised emulsions with an oil content of 10% and protein concentrations below 10% typically behave as Newtonian fluids [33].

Based on Figure 2b, the flow behaviour of the emulsions was characterised using the Ostwald–de Waele model, which fits the shear stress–shear rate relationship. This model provides two key parameters: the flow behaviour index (n), which describes the rheological type of the fluid, and the consistency index (K), which reflects the intrinsic viscosity of the emulsion. As shown in Table 2, the R-squared values ranged from 0.9451 to 1.000, indicating that the model did fit well the experimental data for all samples under investigation.

The results showed that n for HWE was exactly 1.0, confirming its Newtonian fluid nature. By contrast, both NWE and GWE showed n values below 1.0, indicating shear-thinning behaviour, with GWE exhibiting a more pronounced non-Newtonian character. In addition, the K value of GWE was approximately 2-fold and 5-fold higher than that of NWE and HWE, respectively. Generally, in emulsion systems, higher viscosity is associated with improved physical stability [19], suggesting that GWE may offer enhanced stability. The result is also similar with a previous study showing that calcium–heat treatment of WPI-stabilised emulsions can exhibit viscosities up to ten times greater than that of non-heated WPI-stabilised emulsions, although this study [32] used higher oil phase than the one applied here.

### 3.5. Physical Stability

The emulsion capacity, emulsion droplet size, and flow behaviour of these emulsions were then systematically studied after typical food processing (e.g., thermal treatment, acidic pH, and cold storage), in order to assess emulsion stability.

#### 3.5.1. Thermal Stability

**Figure 3:**
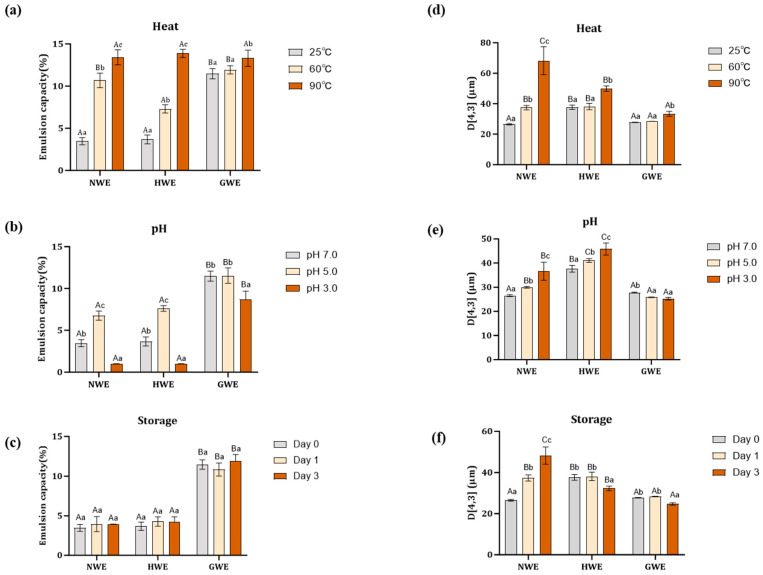
The impact of typical food processing conditions on the emulsion capacity and droplet size. (**a**–**c**) The impact of heat, pH, and storage on the emulsion capacity of emulsions. (**d**–**f**) The impact of heat, pH, and storage on the droplet size. Uppercase letters indicate significant differences between different emulsion groups (NWE, HWE, and GWE) under the same processing treatment condition (i.e., intergroup comparisons, *p* < 0.05). Lowercase letters indicate significant differences within each emulsion group under different processing treatment conditions (i.e., intragroup comparisons, *p* < 0.05).

To evaluate the thermal stability of emulsions, samples were subjected to temperatures of 25 °C, 60 °C, and 90 °C for 30 min. As shown in Figure 3a, GWE consistently exhibited the highest EC values compared to NWE and HWE across all temperature conditions. At 25 °C, the EC of GWE was significantly higher than that of the other two emulsions. As the temperature increased to 60 °C, the EC of NWE and HWE increased markedly. At 90 °C, no significant differences in EC were observed among the three emulsions. While the EC of NWE and HWE changed significantly after heat treatment, that of GWE remained stable, indicating better thermal resistance.

As shown in Figure 3d, heat treatment significantly increased the emulsion droplet size of NWE. At 60 °C, the NWE oil droplet size became comparable to that of HWE and was significantly larger than that of GWE. At 90 °C, NWE showed the largest droplet size, followed by HWE, while GWE maintained the smallest size.

This droplet size increase resulted in an increased flow behaviour n and a decreased consistency index *K* (as shown in Table 2). This phenomenon can likely be attributed to heat-induced unfolding of non-heated whey proteins, leading to more exposed hydrophobic regions and enhanced protein adsorption at the oil interface, which results in an enhanced EC. Meanwhile, the heat-induced unfolding of the proteins may result in protein deformation and aggregation [36,37], leading to emulsion droplet aggregation. Consequently, the emulsion droplet size increased, while the total number of droplets decreased, and the proportion of deformable droplets possibly increased, resulting in a Newtonian flow behaviour with reduced viscosity. This confirms that heat treatment had a significant impact on the flow properties of NWE, altering its rheological behaviour to resemble that of HWE, which exhibited Newtonian characteristics throughout. By contrast, GWE demonstrated the highest thermal stability, maintaining its original shear thinning flow behaviour and high consistency index with minimal changes in both EC and droplet size.

**Figure 4:**
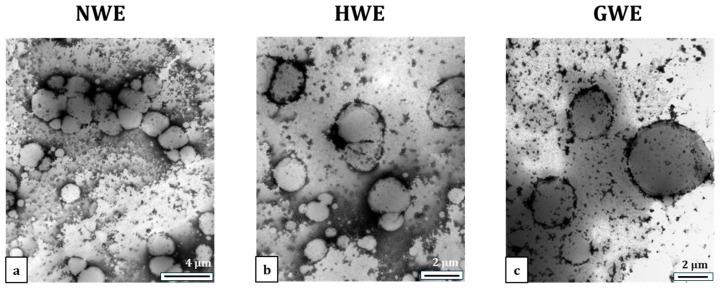
TEM images of emulsions after heat treatment at 90 °C for 30 min: (**a**) NWE, (**b**) HWE, and (**c**) GWE.

TEM was used to observe the morphology of emulsions post heat treatment of 90 °C for 30 min (Figure 4). As shown in Figure 4a, the NWE sample shows severe protein aggregation at the interface and droplet coalescence, indicating significant heat-induced destabilisation. HWE exhibited less aggregation but still showed droplet fusion and irregular protein deposition (Figure 4b), suggesting partial destabilisation. On the other hand, GWE maintained well-defined droplets with minimal aggregation and a uniform protein film (Figure 4c), confirming that the nanogel-based structure effectively prevented heat-induced destabilisation and enhanced emulsion stability. Furthermore, TEM observations also confirmed the presence of heat-induced protein aggregates in the continuous phase of the emulsions. These un-adsorbed protein aggregates, formed as a result of thermal denaturation, are believed to play a critical role in the heat-induced flocculation of emulsion droplets [33]. Specifically, these aggregates may interact with the interfacial protein layers of neighbouring droplets, thereby facilitating droplet coalescence [38]. Moreover, recent studies [39,40] have also demonstrated that un-adsorbed proteins can cause the desorption of adsorbed proteins from the oil–water interface, weakening the interfacial barrier and leading to an increase in droplet size. This highlights the structural destabilisation challenges posed by thermal processing and the importance of enhancing interfacial protein adsorption to maintain emulsion stability.

The superior physical stability of GWE among the tested samples was likely due to its nanogel-based dense physical barrier, which effectively resisted thermal stress as these nanogels have been previously shown to remain structurally stable even under ultra-high temperature (UHT) processing at 140 °C [14], thereby minimising the impact of heat on the emulsion.

#### 3.5.2. pH Stability

To evaluate pH stability, emulsions were adjusted from pH 7 to 5 or 3. As shown in Figure 3b, the emulsion capacity of both NWE and HWE increased significantly at pH 5 but decreased markedly at pH 3. At pH 5, no significant difference was observed between the emulsion capacity of NWE and HWE; however, both exhibited significantly lower EC values than GWE. At pH 3, all three emulsions showed reduced EC values, with those of NWE and HWE reducing to minimal values (below 2%) while that of GWE remaining significantly higher at 8%.

Figure 3e further shows an increase in emulsion droplet size as pH decreased in NWE and HWE. Correspondingly, these changes induced a change in the viscosity of this NWE emulsion (Table 2), with a trend towards higher shear thinning and lower viscosity at pH 5 and a reversed trend at pH 3. These changes can be closely linked to pH-induced protein conformational alteration. At pH 5, near the isoelectric point of WPI, reduced electrostatic repulsion and exposure of hydrophobic regions promote protein–protein interactions and aggregation at the interface, strengthening the interfacial protein network, which increased emulsion capacity and viscosity. By contrast, at pH 3, proteins carry strong positive charges, resulting in enhanced electrostatic repulsion between droplets. This increased electrostatic repulsion further weakened protein adsorption at the O/W interface and disrupted the formation of a cohesive interfacial network [41] within NWE and HWE, contributing to a shift toward Newtonian-like flow behaviour. Although this led to lower viscosity and flow resistance, droplet aggregations still occurred, likely due to insufficient interfacial coverage, which in turn resulted in an increase in droplet size.

By contrast, GWE exhibited much greater stability under pH stress, showing smaller changes in EC, with only a small reduction noticeable at pH 3, and a minimal reduction in emulsion droplet size which resulted in a similarly small reduction in consistency index and small increase in flow behaviour. This stability was likely attributed to the nanogel’s ability to undergo a reversible decrease in zeta potential at pH 5 [14], with no major changes observed in particle size, thereby preserving its interfacial structure and emulsifying functionality despite changes in pH conditions.

#### 3.5.3. Storage Stability

The stability of emulsions during 3-day cold storage at 4 °C was evaluated. Compared with other food processing conditions, cold storage had minimal impact on emulsion capacity across all samples but still led to a significant increase in emulsion droplet size and flow behaviour index, along with a decrease in consistency index for NWE (Table 2). During storage, emulsion stability is influenced by droplet size and gravitational effects according to Stokes’ law. Gravitational forces promote creaming and protein aggregation, which accelerates emulsion droplet aggregation, leading to a gradual increase in droplet size. It is obvious that NWE was the most unstable emulsion of the three, with creaming resulting in larger droplets (Figure 3f) and Newtonian fluid behaviour (Table 2) as a probable reason for this instability.

On the other hand, HWE showed a relative stability in particle size and EC during storage, likely related to the higher viscosity of heat-treated WPI in the continuous phase.

By comparison, GWE showed the least change in EC, droplet size, and flow behaviour among the three emulsions during storage. GWE remained the most stable across all measured parameters, demonstrating superior resistance to time-induced destabilisation. In addition to having a relatively low level of un-adsorbed protein in the continuous phase (~30%), and a higher continuous phase viscosity [14], its stability can also be attributed to its dense and uniform interfacial coverage formed by nanogel-stabilised WPI, which provides full surface protection of emulsion droplets. Such a robust interfacial layer not only prevents droplet coalescence but also enhances resistance against gravitational forces by maintaining droplet integrity and inhibiting further creaming, thereby preserving emulsion structure and rheological properties during storage.

**Figure 5:**
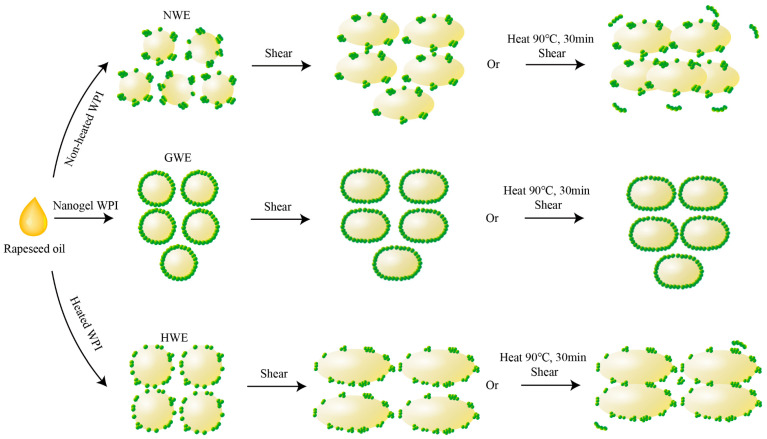
Schematic illustration of flow behaviour reflecting the stability of emulsions before and after food processing conditions.

Therefore, based on the above results, Figure 5 illustrates the proposed stabilisation mechanisms of the three emulsions. In the case of NWE, it is stabilised by non-heated WPI which tends to form partial multilayer coverage with protein clumps at the droplet interface. Although HWE shows some improvement, similar issues remain, as it is stabilised by heat-treated WPI that forms partial multilayer coverage with protein aggregations at the droplet interface. As a result, the emulsion structures of NWE and HWE are more susceptible to disruption under typical food processing conditions. Their flowability increases and their rheological behaviour becomes closer to that of Newtonian fluids, along with droplet aggregation, increased droplet size, and pronounced changes in viscosity. In addition, a large amount of un-adsorbed proteins may aggregate under processing conditions, further compromising emulsion stability.

By contrast, GWE is stabilised by nanogel WPI which forms a full and dense covering film on the droplet surface. Nanogel WPI exhibited superior stability under heat and acidification. Consequently, the combination of full surface coverage for the oil droplets and structurally stable Pickering particles provides GWE with relatively integrated interfacial structures, enabling it to maintain consistent flow behaviour under typical food processing conditions. Droplet aggregation is then minimal, and its droplet size, emulsifying capacity, and viscosity remain highly stable.

## 4. Conclusions

This study made efforts to quantify the impact of protein structure (from non-heated to heated to nanogel formed by heating in the presence of limiting calcium ions) on the capacity of whey protein to stabilise oil in water emulsions.

Systematic comparison of the three emulsions showed that protein adsorption plays a crucial role in emulsion stability, with nanogel WPI offering significantly higher protein adsorption and surface load at the oil–water interface compared to non-heated and heat-treated WPI. Beyond the quantity of protein adsorption, this study further confirmed that emulsion interfacial stability is also closely related to the surface coverage on the oil droplets. Theoretical estimation of the surface coverage of 90% and TEM observations of an almost full monolayered nanogel protein covering the droplet surfaces indicate that nanogel WPI likely forms a hexagonal close-packed arrangement at the oil interface. This dense coverage by nanogel WPI also enhances the viscosity and limits the flow behaviour of the emulsions, which may prevent flocculation, aggregation, and potential phase separation. Consequently, this improves the overall physical stability of the emulsion, such as emulsifying capacity and droplet size, particularly under common food processing conditions, such as acidic pH, heat treatment, and shelf life.

Further investigations are needed to clarify how interfacial features influence emulsion stability during long-term storage and limit potential oil oxidation, as well as how these features affect the nutritional quality of the emulsion, including the digestibility and bio-accessibility of both the oil and the protein.

## Figures and Tables

**Table 1:** Characterisation of emulsions stabilised by non-heated, heated, and nanogel WPI.

Sample	D[4, 3] (μm)	D[3, 2] (μm)	ζ-Potential (mV)	*A* (m^2^/kg)	Protein Adsorption (%)	Γs (mg/m^2^)	Cs	EC (%)
NWE	26.5 ± 0.43 ^a^	12.2 ± 1.16 ^a^	−56.2 ± 0.90 ^a^	556.1 ± 43.90 ^c^	21.8 ± 1.75 ^a^	4.0 ± 0.63 ^a^	63.6 ± 6.05 ^a^	3.5 ± 0.43 ^a^
HWE	37.7 ± 1.42 ^b^	18.0 ± 0.16 ^b^	−52.1 ± 1.59 ^b^	369.8 ± 3.08 ^a^	30.1 ± 1.26 ^b^	8.1 ± 0.41 ^b^	4.0 ± 0.035 ^b^	3.7 ± 0.53 ^a^
GWE	27.8 ± 0.15 ^a^	13.6 ± 0.26 ^a^	−41.7 ± 0.36 ^c^	490.7 ± 6.45 ^b^	68.7 ± 0.11 ^c^	13.6 ± 0.21 ^c^	0.9 ± 0.017 ^c^	11.5 ± 0.60 ^b^

Different superscript letters indicate statistical difference between emulsion samples at *p* < 0.05.

**Table 2:** Effect of food processing conditions on consistency index (*K*) and flow index (*n*) of emulsions.

Treatment	Condition	NWE			HWE			GWE		
		*K*	*n*	R^2^	*K*	*n*	R^2^	*K*	*n*	R^2^
Heat	25 °C	4.0 ± 0.10 ^Bb^	0.73 ± 0.00 ^Ba^	0.9793	1.7 ± 0.06 ^Aa^	1.00 ± 0.00 ^Cb^	0.9939	8.3 ± 0.10 ^Cc^	0.69 ± 0.00 ^Aa^	0.9866
	60 °C	1.6 ± 0.00 ^Aa^	0.93 ± 0.00 ^Bb^	0.9832	1.9 ± 0.13 ^Bb^	0.97 ± 0.01 ^Ca^	0.9924	6.3 ± 0.17 ^Cb^	0.70 ± 0.00 ^Aa^	0.9713
	90 °C	1.8 ± 0.39 ^Aa^	0.91 ± 0.05 ^Bb^	0.9870	1.9 ± 0.13 ^Ab^	0.94 ± 0.02 ^Ba^	0.9911	5.4 ± 0.21 ^Ba^	0.72 ± 0.05 ^Aa^	0.9771
pH	7	4.0 ± 0.10 ^Bb^	0.73 ± 0.00 ^Bb^	0.9793	1.7 ± 0.06 ^Aa^	1.00 ± 0.00 ^Cbc^	0.9939	8.3 ± 0.10 ^Cb^	0.69 ± 0.00 ^Aa^	0.9866
	5	5.3 ± 0.13 ^Bc^	0.65 ± 0.00 ^Aa^	0.9451	2.1 ± 0.26 ^Ab^	0.87 ± 0.02 ^Ca^	0.9891	6.4 ± 0.93 ^Ba^	0.75 ± 0.07 ^Ba^	0.9960
	3	2.1 ± 0.06 ^Aa^	0.87 ± 0.00 ^Bc^	0.9840	2.0 ± 0.21 ^Ab^	0.96 ± 0.05 ^Cb^	0.9911	5.9 ± 0.44 ^Ba^	0.83 ± 0.00 ^Ab^	0.9779
Storage	0 d	4.0 ± 0.10 ^Bc^	0.73 ± 0.00 ^Ba^	0.9793	1.7 ± 0.06 ^Aa^	1.00 ± 0.00 ^Cc^	0.9939	8.3 ± 0.10 ^Cc^	0.69 ± 0.00 ^Aa^	0.9866
	1 d	2.0 ± 0.00 ^Bb^	0.88 ± 0.00 ^Bb^	0.9842	1.8 ± 0.17 ^Aa^	0.98 ± 0.02 ^Cb^	1.0000	6.5 ± 0.10 ^Ca^	0.71 ± 0.00 ^Ab^	0.9846
	3 d	1.6 ± 0.10 ^Aa^	0.93 ± 0.01 ^Bc^	0.9874	2.1 ± 0.06 ^Bb^	0.93 ± 0.00 ^Ba^	0.9925	7.0 ± 0.05 ^Cb^	0.68 ± 0.00 ^Aa^	0.9781

*K* is the consistency index (mPa s^n^); *n* is the flow behaviour index (dimensionless) and describes the divergence from the Newtonian model. Uppercase letters indicate significant differences between different emulsion groups (NWE, HWE, and GWE) under the same processing treatment condition (i.e., intergroup comparisons, *p* < 0.05). Lowercase letters indicate significant differences within each emulsion group under different processing treatment conditions (i.e., intragroup comparisons, *p* < 0.05).

## Data Availability

The original contributions presented in this study are included in the article, and further inquiries can be directed to the corresponding author.

## Abbreviations

The following abbreviations are used in this manuscript: WPI, whey protein isolate; NWE, emulsion stabilised by non-heated WPI; HWE, heat-treated emulsion stabilised by WPI; GWE, emulsion stabilised by nanogel WPI; TEM, transmission electron microscopy; Γs, surface load; *Cs*, surface coverage; EC, emulsion capacity; *η*, viscosity; *K*, consistency index; γ, shear rate; *n* is the flow behaviour index.
